# Projected patterns of land uses in Africa under a warming climate

**DOI:** 10.1038/s41598-024-61035-0

**Published:** 2024-05-29

**Authors:** Ibrahim Yahaya, Runhong Xu, Jian Zhou, Shan Jiang, Buda Su, Jinlong Huang, Jing Cheng, Zhibo Dong, Tong Jiang

**Affiliations:** 1 Research Institute of Climatic and Environmental Governance, Institute for Disaster Risk Management/School of Geographical Science Nanjing, Nanjing University of Information Science and Technology, Nanjing, 210044 China; 2 Department of Geography, Gombe State University, P.M.B, 127, Gombe, Gombe State Nigeria; 3 School of Remote Sensing and Geomatics Engineering, Nanjing University of Information Science and Technology, Nanjing, 210044 China; 4 School of Geographical Science, Qinghai Normal University, Xining, 810008 China; 5 University of Chinese Academy of Sciences, Beijing, 100049 China; 6 School of Geographical Science, Jiangsu Second Normal University, Nanjing, 210013 China

**Keywords:** Environmental sciences, Environmental social sciences

## Abstract

Land-use change is a direct driver of biodiversity loss, projection and future land use change often consider a topical issue in response to climate change. Yet few studies have projected land-use changes over Africa, owing to large uncertainties. We project changes in land-use and land-use transfer under future climate for three specified time periods: 2021–2040, 2041–2060, and 2081–2100, and compares the performance of various scenarios using observational land-use data for the year 2020 and projected land-use under seven Shared Socioeconomic Pathways Scenarios (SSP): SSP1-1.9, SSP1-2.6, SSP2-4.5, SSP3-7.0, SSP4-3.4, SSP4-6.0 and SSP5-8.5 from 2015 to 2100 in Africa. The observational land-use types for the year 2020 depict a change and show linear relationship between observational and simulated land-use with a strong correlation of 0.89 (P < 0.01) over Africa. Relative to the reference period (1995–2014), for (2021–2040), (2041–2060), (2081–2100), barren land and forest land are projected to decrease by an average of (6%, 11%, 16%), (9%, 19%, 38%) respectively, while, crop land, grassland and urban land area are projected to increase by (36%, 58%, and 105%), (4%, 7% and 11%), and (139%, 275% and 450%) respectively. Results show a substantial variations of land use transfer between scenarios with major from barren land to crop land, for the whole future period (2015–2100). Although SSP4-3.4 project the least transfer. Population and GDP show a relationship with cropland and barren land. The greatest conversion of barren land to crop land could endanger biodiversity and have negative effects on how well the African continent's ecosystem’s function.

## Introduction

Land-use change attributed to one of the most important human effects on earth system^1,2^ and most human activities have a direct impact on land use and land cover (LULC)^3^. Utilisation of land resources is referred to as land use, while the type of physical substance covering the Earth’s surface is referred to as land cover^4^. Both (LULC) can be strongly linked with local and regional climate^1,5,6^. In the future, land use activities are likely to expand and/or intensify further to meet growing demands for food, fiber, and energy^7^. However, changes in biogeochemical and biophysical features were responsible driving global, regional, and local land use shifts^8^. Three-quarters of the Earth's land surface has been changed by humans over the last millennium^9,10^, and global land use changes are four times greater than previously estimated^11^. Land use change is essential to successfully address global sustainability challenges such as climate change, biodiversity loss, and food security Since land use change has a significant impact on carbon sources and sinks^12–14^, can lead to habitat loss^15^, and promotes food production^16^.

Land offers a variety of economic, sociological, and ecological objectives as a crucial natural resource that exacerbated by climate change^17^. As a result of social development and environmental factors, land dynamics are continuously changing^18^. In particular, land use changes have been a focal point of debate among researchers especially on the concern of environmental programme^19–22^. Land resources provide a variety of ecosystem services that are essential for human survival on earth^23^. Though, the use of modelling based on the shared socio-economic pathways (SSPs)^24,25^, future land-use change has been investigated^26^. The results show potential future land-use outcomes focused on greenhouse gas emissions, food provision, and price. However, Future changes in land use under a variety of climate models suggest possible effects on biodiversity and ecosystems^27^. Therefore, it is essential to predict future land-use change under various warmer climate scenarios, which are in part driven by climate change. This needs to be investigated into, particularly in Africa.

The Coupled Model Intercomparison Project Phase 6 (CMIP6), which provides information on land use changes, uses the Harmonisation of Global Land-use Change and Management for the period 850–2100 (LUH2)^7^ to present a consistent and comprehensive picture of land-use changes, particularly when dealing with changes on the environment for developing strategies for natural resource management. As a result, they are tasked with producing estimates of future global climate change as part of the Coupled Model Intercomparison Project 6 (CMIP6)^28,29^. To better simulate Earth system dynamics and assess the effects of land use (LU) on climate and biogeochemical cycling, existing Earth system models (ESMs) now routinely contain land use records as a substantial input in the Climate Model Intercomparison Project Phase 6 (CMIP6)^7,30^, the Land Use Model Intercomparison Project (LUMIP) has been established to expand our knowledge of the implications of LU on climate^31^. However, Integrated assessment models (IAMs) are pivotal for evaluating climate objectives, such as those in the Paris Agreement, aiming to limit global warming to below 2° C and pursue 1.5° C above pre-industrial levels^32^. These models combine detailed energy system technologies with simplified economic and climate science models, aiding in exploring various population, economic, and technological pathways for climate mitigation^33^. Depending on inputs and background information, IAMs may consider additional factors like sustainable development goals. Many IAMs prioritize cost-effectiveness, deploying mitigation options globally and sectoral to achieve warming limits at minimal expense. However, the 1.5° C target presents significant challenges for IAMs, pushing their limits^34^. Despite these challenges, IAMs have evolved to encompass a comprehensive range of mitigation measures and technologies, addressing uncertainties in future energy landscapes and complex interconnections between variables^35^.

One of the areas identified as a climate change hotspot is Africa (see AR4 and 5^36,37^), which also experiences adverse climate conditions, threats to human health, and food insecurity^36,38–40^. However, Climate change is already hitting the most vulnerable hardest, and contributing to food insecurity, population displacements and stress on water resources in Africa^8^. Climate models from coupled intercomparison project (CMIP6) demonstrated an increasing warming pattern of potential evapotranspiration in Africa which potentially have far implications for water availability, ecosystem dynamics, and agricultural strategies^41^. According to estimates, warming situations pose the risk of wreaking havoc on food security and crop output^42^. According to the Food and Agriculture Organization of the United Nations^43^, the number of undernourished individuals has increased by 45.6% since 2012 in the sub-Saharan African nations that are prone to drought^44^.

In order to guide the implementation of climate policies in the IAMs, shared climate policy assumptions (SPAs) from (SSP1: Sustainability—Taking the Green road), (SSP2: Middle of the road), (SSP3: Regional rivalry—A rocky road), (SSP4: Inequality—A road divided), and (SSP5: Fossil—fuel development—Taking the highway) have been developed^45^. Most of the work in Africa mainly focuses on land use land cover change focus on remote sensing datasets (LULC)^22,46–48^. While studies on the future land use changes were minimal and focus on some regions within the continent^4^, and land use transfers/conversion and comparing the performance of the African based historical land use with Harmonization datasets (LUH2) were lacking. The studies also concentrate on the application of the expected future land-use scenarios under the SSPs (SSP1-1.9, SSP1-2.6, SSP2-4.5, SSP3-7.0, SSP4-3.4, SSP4-6.0, and SSP5-8.5) and focus on three clearly defined time periods; near-term (2021–2040), mid-term (2041–2060), and long-term (2081–2100), we applied and combined historical land-use data and future land-use data to better understand regional dynamics and transfer/conversion of land-use in Africa under future warming climate change. The most important factors in anticipating and minimising their effects on sustainable land-use management practises in Africa have not been properly examined regarding future land-use projections regarding transfer or conversion from one land-use to another. The study's goal is to project future land-use change on the African continent under future warming climate conditions using LUH2 datasets and assess the relationship with socioeconomic SSPs and how well they perform in comparison to historical land-use data.

## Results

### Correlation between observation land-use and SSPs scenarios

We calculated temporal correlation coefficients in Africa and its various regions concurrently and found 0.89 (P < 0.01) for the observed land-use data for 2020, over Africa, indicating that LUH2 datasets can perform optimally and can simulate the temporal changes and spatial distribution characteristics of land use types in the Africa continents. The original data of LUH2 with 0.25° resolution was used to compare with the observational land-use data from ESA. Additionally, various regions have a substantial correlation coefficient, with r values across NAF, SAH, WAF, CAF, EAF, and SAF, respectively, of 0.86, 0.87, 0.89, 0.86, 0.87, and 0.89 at (P < 0.01) confidence level (shown in Table 1).

**Table 1:** Person’s correlation coefficient between observation land-use of 2020 and SSPs 2020 projection area mean of all (SSPs) in Africa and its Regions. *Significant at 0.01 level.

Regions		SSPs	Observation
Africa	SSPs	1	0.89*
	Observation	0.89*	1
Northern Africa (NAF)	SSPs	1	0.86*
	Observation	0.86*	1
Sahara (SAH)	SSPs	1	0.87*
	Observation	0.87*	1
Western Africa (WAF)	SSPs	1	0.85*
	Observation	0.89*	1
Central Africa (CAF)	SSPs	1	0.86*
	Observation	0.86*	1
Eastern Africa (EAF)	SSPs	1	0.87*
	Observation	0.87*	1
Southern Africa (SAF)	SSPs	1	0.89*
	Observation	0.89*	1

### Changes in historical land-use in Africa

**Figure 1:**
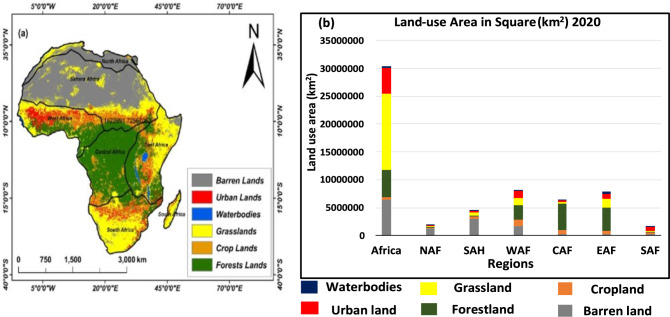
Spatial distribution of land use types in 2020 (**a**) and (**b**) land use Areal types in square kilometres (km^2^) across Africa and its Regions.

**Figure 2:**
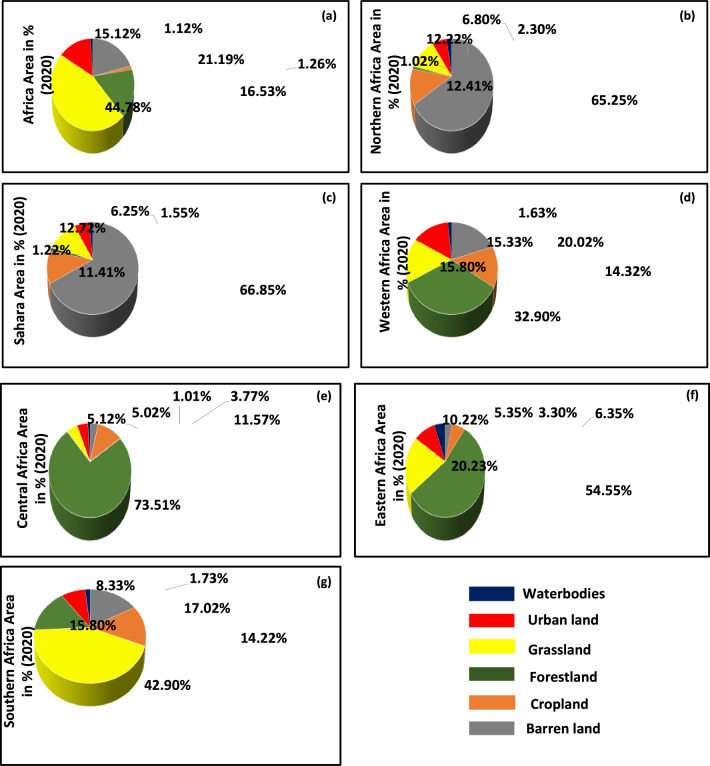
Areal percentages changes in land use for the observational period (2020), over (**a**) Africa, (**b**) Northern Africa, (**c**) Sahara, (**d**) Western Africa, (**e**) Central Africa, (**f**) Eastern Africa and (**g**) Southern Africa for Waterbodies (Blue), Urban land (Red), Grassland (Yellow), Forestland (Geen).

In the year 2020, we identified that grassland was the dominant land use type in the region, accounted for 44.78% of the total area. It was primarily distributed in the southern, eastern, and western regions of the continent as well as in some areas of the northern and Saharan regions. The second was barren land, accounted for 21.19% of the total area, it was mostly found in the Northern and Sahara region. The third was forest land, made up 16.53% of the total area and was most prevalent in the Central region. The Urban land accounted for 15.12% mainly dominated within the Western region of Africa. The cropland had made of 1.26% mainly distributed across all the regions and dominated within the Western area. The water bodies which accounted for 1.12% mainly dominated in Eastern region. The crop land in the region were scarce, likewise, urban land and waterbodies were dominant in Western (WAF) and Eastern region (EAF) respectively (Figs. 1 and 2a). However, the land use type over Northern region (NAF) and Sahara (SAH) was dominated by barren land accounted for (65.25%) and (66.85%) respectively, the cropland in NAF and SAH accounted for 12.41% and 11.41% respectively. Forestland were marginal in both NAF and SAH regions accounted for 1.02% and 1.22% respectively. Grassland accounted for 12.22% and 12.72% over NAF and SAH respectively. The urban land accounted for 6.80% and 6.25% respectively over NAF and SAH. Waterbodies appears scarce in both the two regions accounted for 6.80% and 6.25% respectively (Figs. 1 and 2b,c). However, Western region (WAF) accounted for 20.02%, 14.32%, 32.90%, 15.80%, 15.33%, and 1.63% respectively, for barren land, cropland, forest land, grassland, urban land, and waterbodies respectively (Figs. 1 and 2d). Likewise, Central region (CAF), accounted for 3.77%, 11.57%, 73.51%, 5.12%, 5.02%, and 1.01% for barren land, cropland, forest land, grassland, urban land, and waterbodies respectively (Figs. 1 and 2e).

The Eastern region (EAF), accounted for 3.30%, 6.35%, 54.55%, 20.23%, 10.22%, and 5.35% respectively, for barren land, cropland, forest land, grassland, urban land, and waterbodies (Figs. 1 and 2f). Moreover, Southern region (SAF), accounted for 17.02%, 14.22%, 15.80%, 42.90%, 8.33%, and 1.73% for barren land, cropland, forest land, grassland, urban land, and waterbodies respectively (Figs. 1 and 2g).

### Projected changes in land-use in Africa

**Figure 3:**
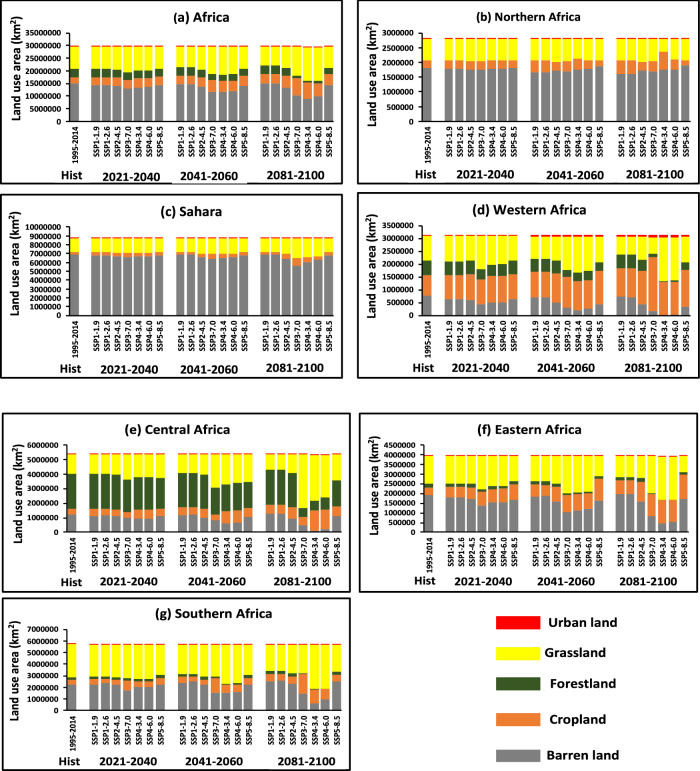
Changes in land use area for the historical baseline period (1995–2014) and projections (2015–2100) averaged over (**a**) Africa, (**b**) Northern Africa, (**c**) Sahara, (**d**) Western Africa, (**e**) Central Africa, (**f**) Eastern Africa and (**g**) Southern Africa for Urban land (Red), Grassland (Yellow), Forestland (Geen), Cropland (Orange), and Barren land (Light Dark), for near-term (2021–2040), mid-term (2041–2060) and long-term (2081–2100) under SSP1-1.9, SSP1-2.6, SSP2-4.5, SSP3-7.0, SSP4-3.4, SSP4-6.0, and SSP5-8.5 Scenarios.

The historical period (1995–2014), the areas of barren land, crop land, forest land, grass land, and urban land in African continent were approximately 1490** × **10^4^ km^2^, 244** × **10^4^ km^2^, 344** × **10^4^ km^2^, 884** × **10^4^ km^2^, and 4.32** × **10^4^ km^2^, respectively (Fig. 3a). However, the changes were calculated relative to the historical period under three period and was found out that barren land is expected to decrease except for SSP1-1.9 and SSP1-2.6 shown a mild increase, the areas of urban land and crop land area is projected to increase in all SSPs, with rapid increase in urban land under SSP4-3.4 and SSP4-6.0 reaching 28** × **10^4^ km^2^ accounted for 600%, and cropland shown a rapid increase under SSP3-7.0 at the end of twenty-first century reaching 674** × **10^4^ km^2^ accounted for 176%, the forest land were projected to decrease in all SSPs, the grassland were shown with the decrease in SSP1-1.9 and SSP1-2.6 and depict an increase under SSP3-7.0, SSP4-3.4, SSP4-6.0 and SSP5-8.5.

The near-term period (2021–2040), the barren land area is projected to be approximately 1388** × **10^4^ km^2^ in all SSPs accounted for reduction of 6% compared to historical period (1995–2014), the crop land area is approximately 334** × **10^4^ km^2^ in all SSPs and was projected to be accounted for an increase of 36% relative to historical period, the forestland area was anticipated to reduce by an area approximately 312** × **10^4^ km^2^ accounted for 9% relative to the historical period, the grass land area is approximately 922** × **10^4^ km^2^ in all SSPs accounted for an increase in 4% compared to the historical period (1995–2014). Likewise, the urban land area is approximately 9.7** × **10^4^ km^2^ was anticipated to increase by 139% relative to the historical period (Fig. 3a).

The mid-term (2041–2060), the barren land area is projected to be approximately 1324** × **10^4^ km^2^ in all SSPs accounted for reduction of 11% compared to historical period (1995–2014), the crop land area is approximately 396** × **10^4^ km^2^ in all SSPs and was projected to be accounted for an increase of 58% relative to historical period, the forestland area was anticipated to reduce by an area approximately 278** × **10^4^ km^2^ accounted for 19% relative to the historical period, the grass land area is approximately 955** × **10^4^ km^2^ in all SSPs accounted for an increase in 7.9% compared to the historical period (1995–2014). Likewise, the urban land area is approximately 15.5** × **10^4^ km^2^ was anticipated to increase by 275% relative to the historical period (Fig. 3a).

The long-term (2081–2100), saw a projected remarkable reduction of the barren land area to be approximately 1243** × **10^4^ km^2^ in all SSPs accounted for reduction of 16% compared to historical period (1995–2014), while crop land area shown with a robust increase of approximately 500** × **10^4^ km^2^ in all SSPs and was projected to be accounted for an increase of 105% relative to historical period, the forestland area was anticipated to reduce by an area approximately 213** × **10^4^ km^2^ accounted for 38% relative to the historical period, the grass land area is approximately 988** × **10^4^ km^2^ in all SSPs accounted for an increase in 11% compared to the historical period (1995–2014). Likewise, the urban land area is approximately 22.2** × **10^4^ km^2^ was anticipated to increase by 450% relative to the historical period (Fig. 3a).

However, comparison was made to various distinct region across the continent, The changes present insights into the land-use changes across different periods in Africa, the Northern Africa (NAF) in the period (2021–2040), (2041–2060), (2081–2100), relative to the historical period 180** × **10^4^ km^2^, the barren land area is approximately 177** × **10^4^ km^2^, 173** × **10^4^ km^2^, 172** × **10^4^ km^2^, denoting for a reduction of 1.7%, 3.9%, 4.4% respectively, over NAF (Fig. 3b), and 674** × **10^4^ km^2^, 666** × **10^4^ km^2^, and 642** × **10^4^ km^2^, denoting a reduction of 2.2%, 3.4%, and 6.9% respectively, over SAH compared to historical period 690** × **10^4^ km^2^. The crop land area is projected to be an increase by 29** × **10^4^ km^2^, 33** × **10^4^ km^2^, 38** × **10^4^ km^2^, accounting of 6%, 21%, 39% respectively, over NAF (Fig. 3b), compared to historical 27** × **10^4^ km^2^. Likewise, the crop land under (SAH) was also projected to be an increase by 37** × **10^4^ km^2^, 41** × **10^4^ km^2^, and 49** × **10^4^ km^2^, accounting of 31%, 44%, and 75% respectively, over NAF relative to historical 28** × **10^4^ km^2^ (Fig. 3b), compared to historical 27** × **10^4^ km^2^. The forest land in both (NAF) and (SAH) were zero % in both the historical and projections (Fig. 3b,c). The grass land area is projected to decrease by 73** × **10^4^ km^2^, 72** × **10^4^ km^2^, 68** × **10^4^ km^2^, accounting for 0.9%, 0.7%, 5.7% respectively, over NAF (Fig. 3b), compared to historical 72** × **10^4^ km^2^. Likewise, the grass land under (SAH) was also projected to be an increase by 158** × **10^4^ km^2^, 163** × **10^4^ km^2^, and 177** × **10^4^ km^2^, accounting for 3%, 6%, and 15% respectively, over NAF relative to historical 153** × **10^4^ km^2^ (Fig. 3c). The urban land area in both (NAF) and (SAH) was anticipated for an increase reaching to 1.96** × **10^4^ km^2^ and 2.2** × **10^4^ km^2^ at the end of twenty-first century, relative to the historical period 0.7** × **10^4^ km^2^ and 0.30** × **10^4^ km^2^ over NAF and SAH respectively. (Fig. 3b,c).

The Western Africa (WAF) in the period (2021–2040), (2041–2060), (2081–2100), relative to the historical period 78** × **10^4^ km^2^, the barren land area is approximately 73** × **10^4^ km^2^, 44** × **10^4^ km^2^, 34** × **10^4^ km^2^, denoting for a reduction of 6%, 43%, 55% respectively, over WAF (Fig. 3d), and 105** × **10^4^ km^2^, 94** × **10^4^ km^2^, and 78** × **10^4^ km^2^, denoting a reduction of 14%, 24%, and 36% respectively, over CAF compared to historical period 123** × **10^4^ km^2^ (Fig. 3e). The crop land area is projected to be an increase by approximately 98** × **10^4^ km^2^, 112** × **10^4^ km^2^, 137** × **10^4^ km^2^, accounting of 25%, 43%, 76% respectively, over WAF compared to historical 78** × **10^4^ km^2^ (Fig. 3d). Likewise, the crop land under (CAF) was also projected to be an increase by 51** × **10^4^ km^2^, 61** × **10^4^ km^2^, and 84** × **10^4^ km^2^, accounting of 40%, 72%, and 84% respectively, over CAF relative to historical 35** × **10^4^ km^2^ (Fig. 3e), The forest land area is projected to decrease by approximately 48** × **10^4^ km^2^, 40** × **10^4^ km^2^, 28** × **10^4^ km^2^, accounting of 14%, 28%, 50% respectively, over WAF compared to historical 58** × **10^4^ km^2^ (Fig. 3d). Likewise, the forestland under (CAF) was also projected to decrease by 226** × **10^4^ km^2^, 205** × **10^4^ km^2^, and 157** × **10^4^ km^2^, accounting of 6%, 14%, and 35% respectively, over CAF relative to historical 241** × **10^4^ km^2^ (Fig. 3e), The grass land area is projected to increase by 105** × **10^4^ km^2^, 110** × **10^4^ km^2^, 104** × **10^4^ km^2^, accounting for 8%, 12%, 6% respectively, over WAF (Fig. 3d), compared to historical 97** × **10^4^ km^2^. Likewise, the grass land under (CAF) was also projected to be an increase by 151** × **10^4^ km^2^, 172** × **10^4^ km^2^, and 212** × **10^4^ km^2^, accounting for 11%, 27%, and 56% respectively, over CAF relative to historical 135** × **10^4^ km^2^ (Fig. 3e). The urban land area in both (WAF) and (CAF) was anticipated for a robust increase reaching to 7.09** × **10^4^ km^2^ and 3.90** × **10^4^ km^2^ at the end of twenty-first century, relative to the historical period 1.28** × **10^4^ km^2^ and 0.53** × **10^4^ km^2^ over WAF and CAF respectively (Fig. 3d,e).

The Eastern Africa (EAF), and the Southern Africa (SAF), also shows a reduction of barren land area, relative to the historical period 190** × **10^4^ km^2^, the barren land area is approximately 163** × **10^4^ km^2^, 146** × **10^4^ km^2^, 130** × **10^4^ km^2^, respectively, over EAF (Fig. 3f), and 205** × **10^4^ km^2^, 199** × **10^4^ km^2^, and 184** × **10^4^ km^2^, respectively, over SAF compared to historical period 226** × **10^4^ km^2^ (Fig. 3g). The crop land area is projected to increase by approximately 65** × **10^4^ km^2^, 80** × **10^4^ km^2^, 101** × **10^4^ km^2^, respectively, over EAF compared to historical 38** × **10^4^ km^2^ (Fig. 3e). Likewise, the crop land under (SAF) was also projected to increase by 51** × **10^4^ km^2^, 65** × **10^4^ km^2^, and 89** × **10^4^ km^2^, respectively, relative to historical 36** × **10^4^ km^2^ (Fig. 3g). The forest land area is projected to decrease by approximately 14** × **10^4^ km^2^, 12** × **10^4^ km^2^, 10** × **10^4^ km^2^, respectively, over EAF compared to historical 20** × **10^4^ km^2^ (Fig. 3e). Likewise, the forestland under (SAF) was also projected to decrease by 22** × **10^4^ km^2^, 19** × **10^4^ km^2^, and 16** × **10^4^ km^2^, respectively, relative to historical 26** × **10^4^ km^2^ (Fig. 3g).

The grass land area is projected to increase by 149** × **10^4^ km^2^, 152** × **10^4^ km^2^, 149** × **10^4^ km^2^, respectively, over EAF (Fig. 3e), compared to historical 144** × **10^4^ km^2^. Likewise, the grass land under (SAF) was also projected to decrease by 284** × **10^4^ km^2^, 284** × **10^4^ km^2^, and 276** × **10^4^ km^2^, respectively, relative to historical 281** × **10^4^ km^2^ (Fig. 3g). The urban land area in both (EAF) and (SAF) was projected to increase reaching to 3.37** × **10^4^ km^2^ and 3.61** × **10^4^ km^2^ at the end of twenty-first century, relative to the historical period 0.38** × **10^4^ km^2^ and 1.03** × **10^4^ km^2^ over EAF and SAF respectively (Fig. 3e,g).

### Dynamic transfer process in future land-use

**Figure 4:**
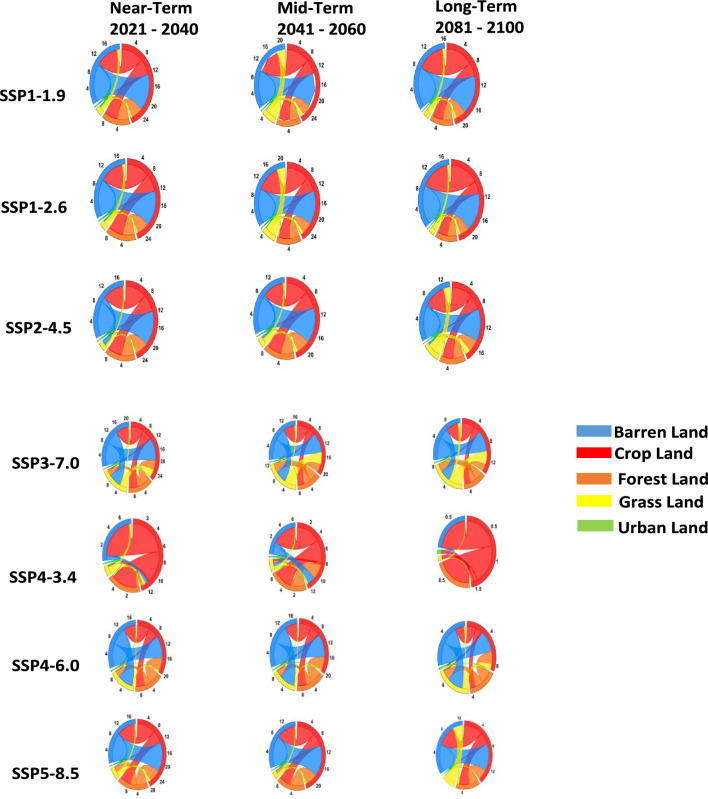
Land use transfers predicted for near-term (2021–2040), mid-term (2041–2060) and long-term (2081–2100) under SSP1-1.9, SSP1-2.6, SSP2-4.5, SSP3-7.0, SSP4-3.4, SSP4-6.0, and SSP5-8.5 Scenarios. The Urban land (Green), Grassland (Yellow), Forestland (Orange), Cropland (Red), and Barren land (Blue). Note: The colour of the arc represents the land use type, the colour of the chord represents the land use transfer type, and the width of the chord represents the land use transfer amount.

According to the future analysis of land-use transfer, the data suggest a rather abrupt change of decreasing rates of barren land use to cropland use which is most evident in Africa (see Fig. 4). We hypothesize that the transition change from barren land to cropland is related to market developments in the context of the global economic and food crisis 2007–2009, followed by crop to barren, forest to crop, and other reciprocal transfers of grass to crop, are the main land-use transfers in Africa (Fig. 4). Urban land transfers were minimal.

The near-term period (2021–2040), the transfer area of barren land to crop land will be approximately 0.5 **× **10^4^ km^2^ to 10 **× **10^4^ km^2^ under all SSPs, with highest transfer of approximately 10 **× **10^4^ km^2^ in both (SSP1-1.9 and SSP5-8.5), and the smallest was under SSP4-3.4, 0.5 **× **10^4^ km^2^, with an average of approximately 8.0 **× **10^4^ km^2^. The transfer area of forest to crop land will be approximately 0.3 **× **10^4^ km^2^ to 4.7 **× **10^4^ km^2^ under all SSPs, with highest transfer of approximately 4.7 **× **10^4^ km^2^ in (SSP5-8.5), and the smallest was under SSP4-3.4, 0.3 **× **10^4^ km^2^, with an average of approximately 3.5 **× **10^4^ km^2^. Moreover, transfer area of barren land to grass land will be approximately 0.2 **× **10^4^ km^2^ to 5.7 **× **10^4^ km^2^ under all SSPs, with highest transfer of approximately 5.7 **× **10^4^ km^2^ in (SSP4-6.0), with an average of approximately 1.9 **× **10^4^ km^2^ (Fig. 4).

Interestingly, the land-use transfer on the Africa continent during mid-term (2041–2060), were still consistent to that of near-term, as barren land to crop land will be approximately 1.3 **× **10^4^ km^2^ to 10 **× **10^4^ km^2^ under all SSPs, with highest transfer of approximately 10 **× **10^4^ km^2^ in both (SSP1-1.9 and SSP1-2.6), and the smallest was under SSP4-3.4, 1.3 **× **10^4^ km^2^, with an average of approximately 7.5 **× **10^4^ km^2^. The transfer area of crop land to forest land will be approximately 2.8 **× **10^4^ km^2^ to 4.2 **× **10^4^ km^2^ under all SSPs, with highest transfer of approximately 4.7 **× **10^4^ km^2^ in (SSP1-1.9, SSP1-2.6, SSP2-4.5), and the smallest was under SSP3-7.0, 2.8 **× **10^4^ km^2^, with an average of approximately 3.7 **× **10^4^ km^2^. The minimal transfer between barren land to grass land were seen with an average of approximately 1.5 **× **10^4^ km^2^. Thereby, grass land to crop land also exhibit a minimal transfer with an average of approximately 1.1 **× **10^4^ km^2^ (Fig. 4).

At the end of the twenty-first century, long-term (2081–2100), the transfer area of barren land to crop land will be approximately 0.0 **× **10^4^ km^2^ to 9 **× **10^4^ km^2^ under all SSPs, with highest transfer of approximately 9 **× **10^4^ km^2^ in both (SSP1-2.6), with an average of approximately 5.3 **× **10^4^ km^2^. The transfer area of forest to crop land will be approximately 0.7 **× **10^4^ km^2^ to 3.0 **× **10^4^ km^2^ under all SSPs, with highest transfer of approximately 3.0 **× **10^4^ km^2^ in (SSP1-2.6), with an average of approximately 2.1 **× **10^4^ km^2^.

### Spatial changes in the future transfer of barren land to cropland in Africa

**Figure 5:**
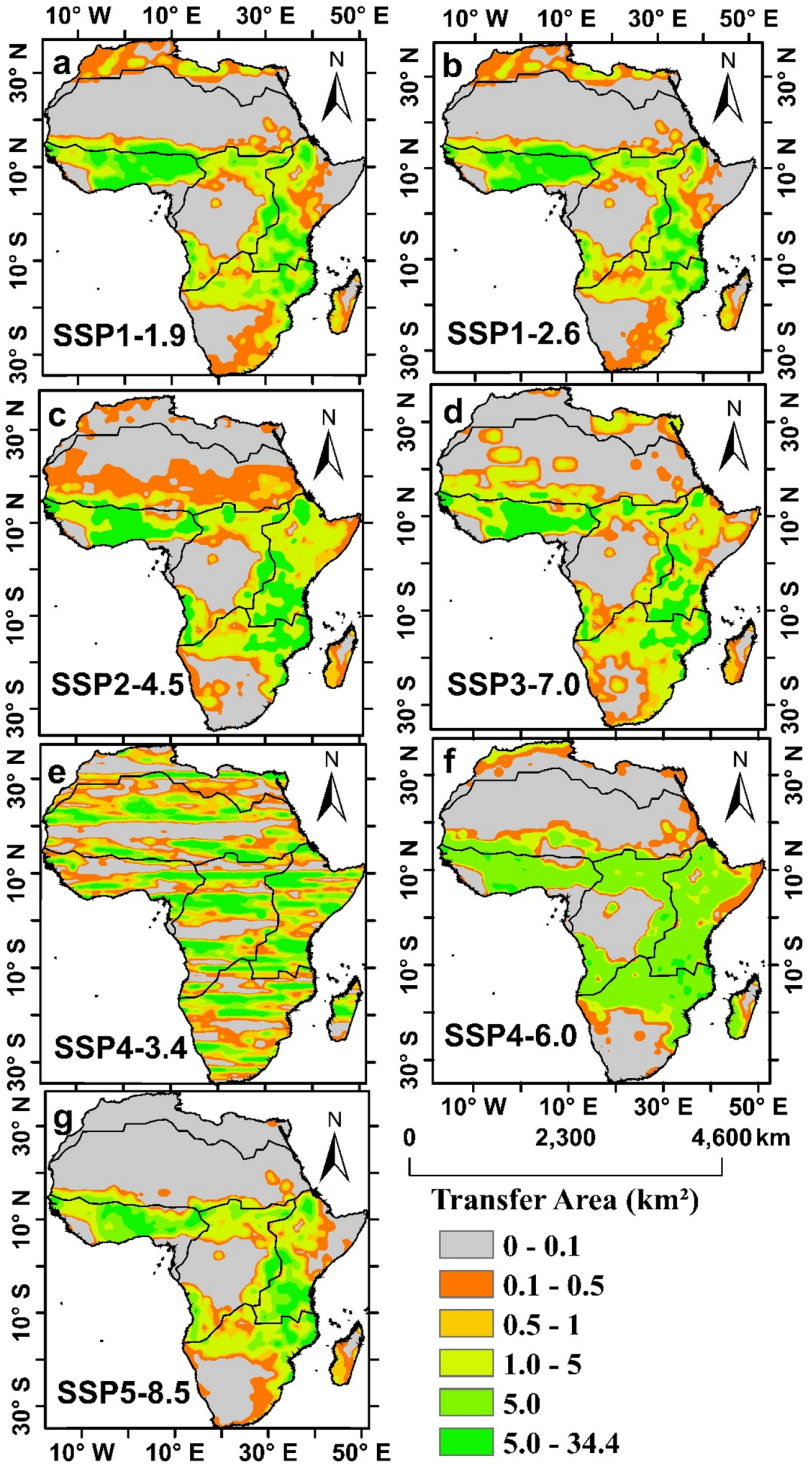
Spatial changes in the areas transferred in (km^2^) from barren land to crop land under different SSPs Scenarios (**a**) SSP1-1.9, (**b**) SSP1-2.6, (**c**) SSP2-4.5, (**d**) SSP3-7.0, (**e**) SSP4-3.4, (**f**) SSP4-6.0 and (**g**) SSP5-8.5 from (2015–2100) at the end of twenty-first century.

Future land-use patterns in Africa show a significant conversion of barren land to cropland, which will represent the largest transfer area in comparison to the historical period (1995–2014), which can be observed in the strong decline in the rate of land use change in most regions of Africa. Furthermore, land degradation, caused by both climatic variability and human activities, has often been associated with cropland abandonment, subsequent expansion of agricultural land and deforestation elsewhere, as widely observed in tropical regions^5^. Our analysis went further to examine the regional variations in the conversion of barren land to cropland across the region, and the results show that the Western (WAF), Eastern (EAF), and a portion of the Southern (SAF) regions will be the ones where this conversion will be most pronounced (Fig. 5). Approximately 201** × **10^4^ km^2^, 199** × **10^4^ km^2^, 167** × **10^4^ km^2^, 140** × **10^4^ km^2^, 12** × **10^4^ km^2^, 137** × **10^4^ km^2^ and 162** × **10^4^ km^2^ have changed between 2015 to 2100 under SSP1-1.9, SSP1-2.6, SSP2-4.5, SSP3-7.0, SSP4-3.4, SSP4-6.0 and SSP5-8.5, respectively. However, SSP4-3.4. in (Fig. 5e) predicts the lowest conversion in the future.

### Spatial changes in the future transfer of forestland to cropland in Africa

**Figure 6:**
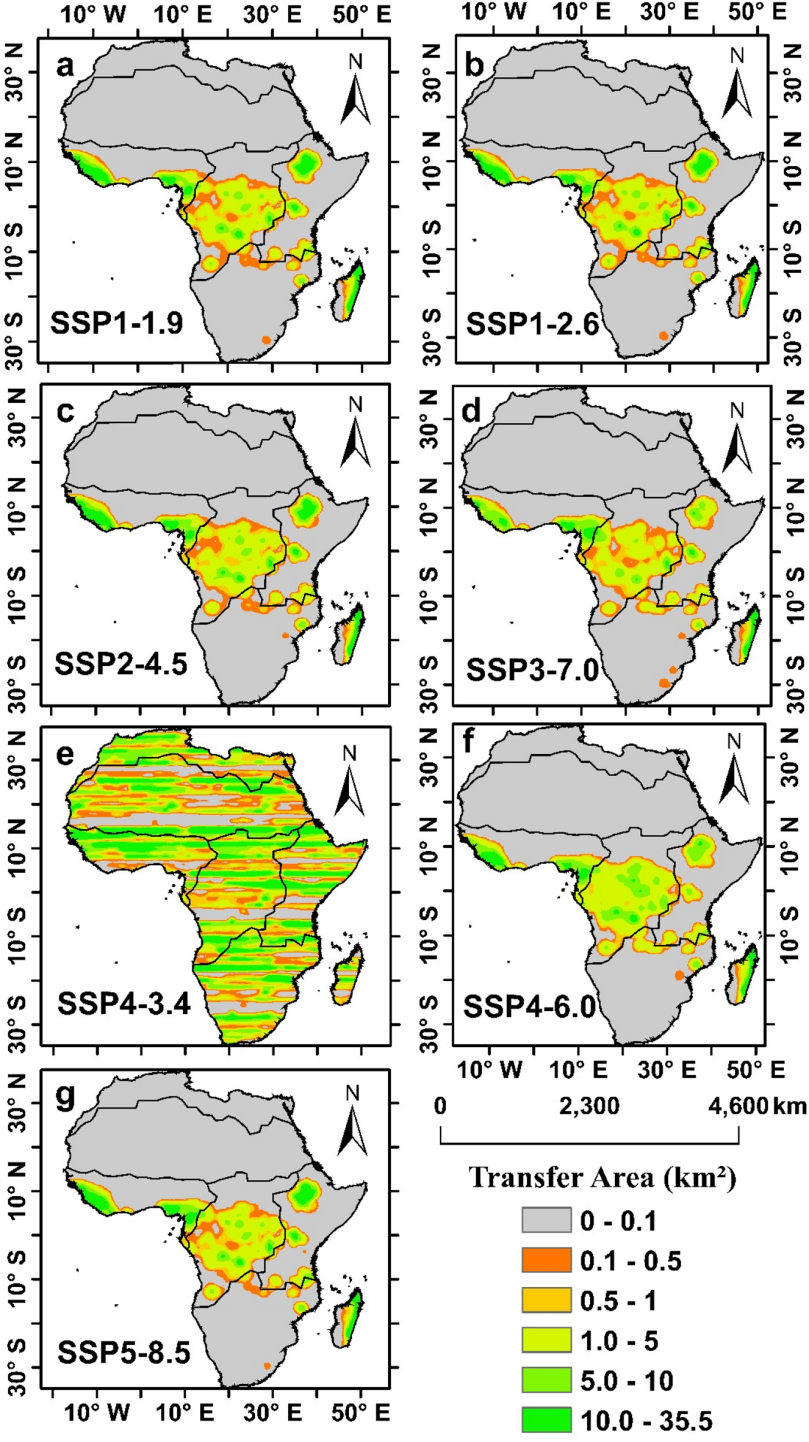
spatial changes in the areas transferred in (km^2^) from forestland to cropland under different SSPs Scenarios (**a**) SSP1-1.9, (**b**) SSP1-2.6, (**c**) SSP2-4.5, (**d**) SSP3-7.0, (**e**) SSP4-3.4, (**f**) SSP4-6.0 and (**g**) SSP5-8.5 from (2015–2100) at the end of twenty-first century.

The spatial pattern of the future land-use changes in Africa exhibits large conversion from forest land to crop land, and the shift will be largest transfer area in the future relative to historical period (1995–2014). Notably, the expansion of cropland led to the most significant land use characteristic on the African continent. Our analysis went further to examine the regional variations in the conversion of forest to cropland across the region, and the results show that the Western (WAF), Central (CAF), and Eastern (EAF) regions will dominate the conversion of forest to crop land (Fig. 6). The changes from 2015 to 2100 under SSP1-1.9, SSP1-2.6, SSP2-4.5, SSP3-7.0, SSP4-3.4, SSP4-6.0, and SSP5-8.5 witness an increase over time and is approximately 68** × **10^4^ km^2^, 68** × **10^4^ km^2^, 70** × **10^4^ km^2^, 56** × **10^4^ km^2^, 14** × **10^4^ km^2^, 75** × **10^4^ km^2^ and 71** × **10^4^ km^2^ respectively (Fig. 6).

### Future changes in population and GDP

**Figure 7:**
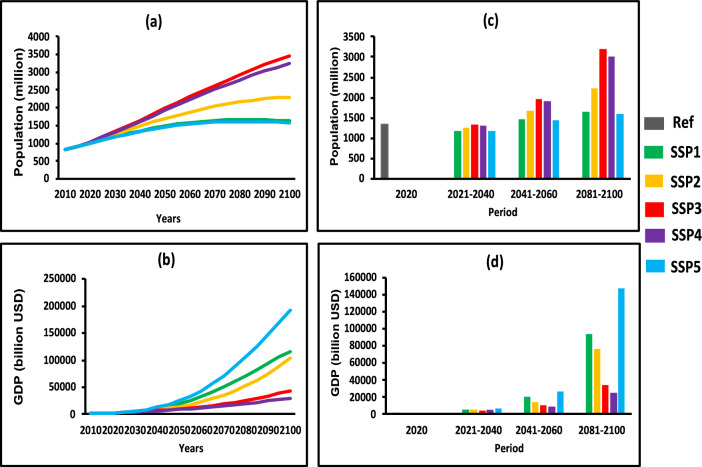
Temporal changes in population (**a**), GDP (**b**), relative changes in population (**c**) and GDP (**d**) for reference period (2020), near-term (2021–2040), mid-term (2041–2060) and long-term (2081–2100) over Africa. Note: the population is in (million), and GDP is in (billion USD).

We also investigate the socioeconomic aspects of land usage in Africa considering predicted GDP and population pathways (Fig. 7). In all scenarios, the population under SSPs increased noticeably, with the lowest increases occurring under SSP1 and SSP5 and the highest increases occurring under SSP3 and SSP4 (Fig. 7a). The GDP grows under SSPs in all scenarios, with the lowest growth rates under SSP3 and SSP4 and the strongest growth rates under SSP1 and SSP5 (see Fig. 7b). The observation population period of (2020) was approximately 1360.68 million. But the projected population under the near-term (2021–2040) is projected to decrease by approximately 1249.38 million (8.1%) compared to the reference period; however, the projected population under the mid-term (2041–2060) increased by approximately 1698.30 million (24.8%); similarly, the long-term period (2081–2100) was projected to increase by approximately 2341.89 million (72.1%).

Relative to the reference period (2020), the GDP is approximately 2414.59 billion USD. The GDP is projected to increase significantly over all time periods for the near term (2021–2040), mid-term (2041–2060), and long term (2081–2100), which is approximately 131.19 billion USD (1.3%), 575.85 billion USD (5.75%), and 3031.06 billion USD (30.31%) respectively, compared to the reference period Fig. 7d.

The correlation of future socioeconomic variables (population and GDP) with crop land and barren land areas (Table 2) showed that population and GDP were significantly correlated with crop land and barren land areas. The result depicts that between population and crop land under near-term (2021–2040), mid-term (2041–2060) and long-term (2081–2100) exhibits a positive correlation in all SSPs (SSP1, SSP2, SSP3, SSP4 and SSP5) scenarios. However, from our analysis in Table 2, showed that strong positive correlation exhibits between GDP and crop land over Africa as indicated under all periods and all SSPs, from near-term to the end of the twenty-first century.

**Table 2:** Correlation of socioeconomic variables (population and GDP) with the cropland and barren land areas (**Significant at 0.05 level**).

Indicators	SSPs	2021–2040	2041–2060	2081–2100
Population vs. Cropland	SSP1	0.97	0.99	0.98
	SSP2	0.99	0.99	0.99
	SSP3	0.99	0.99	0.99
	SSP4	0.99	0.99	0.99
	SSP5	0.96	0.99	0.71
GDP vs. Cropland	SSP1	0.98	0.97	0.87
	SSP2	0.99	0.98	0.98
	SSP3	0.99	0.99	0.99
	SSP4	0.99	0.99	0.99
	SSP5	0.97	0.94	0.89
Population vs. Barren land	SSP1	− 0.67	0.99	− 0.91
	SSP2	− 0.99	− 0.99	0.90
	SSP3	− 0.98	− 0.99	− 0.99
	SSP4	− 0.99	− 0.99	− 0.99
	SSP5	− 0.99	− 0.99	− 0.67
GDP vs. Barren land	SSP1	− 0.60	0.98	0.67
	SSP2	− 0.98	− 0.97	0.96
	SSP3	− 0.98	− 0.99	− 0.99
	SSP4	− 0.99	− 0.99	− 0.99
	SSP5	− 0.94	− 0.97	0.87

Interestingly, the result depicts that between population and barren land under near-term (2021–2040), mid-term (2041–2060) and long-term (2081–2100) exhibits an inverse negative correlation in all SSPs (SSP1, SSP2, SSP3, SSP4 and SSP5) except for SSP1 (0.99) in mid-term (2040–2060) scenarios. However, from our analysis in Table 2, showed that negative correlation exhibits between GDP and barren land over Africa as indicated under all periods and all SSPs, but showed with a positive correlation in SSP1 near-term and long-term (0.98) (0.67) respectively, and long-term SSP2 (0.96) and SSP5 (0.87) at the end of the twenty-first century.

## Conclusions and discussion

This study use observation land use, historical and projections simulation land use under various SSPs to project changes and conversion of land-use patterns in Africa and its sub-regions, and their relationships with socioeconomic changes (population and GDP) in different time scales, utilizing the most recent socioeconomic pathways from LUH2 project, and potential future socioeconomic conditions described in the Shared Socio-economic pathways SSPs^49^, (SSP1-1.9, SSP1-2.6, SSP2-4.5, SSP3-7.0, SSP4-3.4, SSP4-6.0, and SSP5-8.5) to project future land-use changes^45^. It was discovered that LUH2 performed better and can simulate future land-use change after the study utilized a linear relationship to detect the performance of the Simulation datasets with observational land-use. The analysis made use of observational data for 2020, and it also applied this methodology to LUH2 data in the future (from 850 to 2100). To provide a baseline, the years 1995 to 2014 were chosen as the reference period. The study estimated projected land-use change over three distinct time periods to evaluate the region’s response to future climate change. The main conclusions of the study are as follows:

i. The study discovered a 0.89 (P < 0.01) correlation between the observation land-use data for 2020 and the simulation land-use, showing that LUH2 datasets can simulate land use types of temporal changes and spatial distribution characteristics with the greatest accuracy. The observational land-use type in Africa for the year 2020 comprises of barren land, crop land, forest land, grassland, urban land, and waterbodies. Between these land-uses, grassland was the largest with the year average of approximately 1359** × **10^4^ km^2^, which accounted for 44.78%, the second was barren land, with an annual average area of approximately 643** × **10^4^ km^2^, accounted for 21.19%, followed by forest land had an average area of approximately 502** × **10^4^ km^2^, accounted for 16.53%, then Urban land with annual average of approximately 459** × **10^4^ km^2^, accounted for 15.12%, the smallest were cropland and waterbodies with year annual average area of approximately 38** × **10^4^ km^2^, and 33** × **10^4^ km^2^ accounted for 1.26%, and 1.12% respectively.
ii. The future land use changes under near-term (2021–2040), for African continent was dominated by changes in barren land, forest land and crop land, the barren land area will occupy an area of approximately 1291** × **10^4^ km^2^ to 1452** × **10^4^ km^2^ (averaging 1388** × **10^4^ km^2^) as a reduction of 6% relative to the historical period (1995–2014). The forest land occupies an area of approximately 293** × **10^4^ km^2^ to 332** × **10^4^ km^2^ (averaging 312** × **10^4^ km^2^) denoting a reduction of 9% relative to the historical period (1995–2014). Likewise, the crop land occupies an area of approximately 297** × **10^4^ km^2^ to 354** × **10^4^ km^2^ (averaging 334** × **10^4^ km^2^) denoting an increase of 36% relative to the historical period (1995–2014). In respect to land-use transfer, the conversion from barren to crop land being the largest over the region with area approximately 0.1** × **10^4^ km^2^ to 44** × **10^4^ km^2^ (averaging 26** × **10^4^ km^2^ in all SSPs).
iii. The future land use changes under mid-term (2041–2060), for African continent was consistent to near-term as dominated barren land, forest land and crop land, the barren land area will occupy an area of approximately 1172** × **10^4^ km^2^ to 1483** × **10^4^ km^2^ (averaging 1324** × **10^4^ km^2^) as a reduction of 11% relative to the historical period (1995–2014). The forest land occupies an area of approximately 234** × **10^4^ km^2^ to 332** × **10^4^ km^2^ (averaging 312** × **10^4^ km^2^) denoting a reduction of 19% relative to the historical period (1995–2014). Likewise, the crop land occupies an area of approximately 320** × **10^4^ km^2^ to 473** × **10^4^ km^2^ (averaging 394** × **10^4^ km^2^) denoting an increase of 58% relative to the historical period (1995–2014). In respect to land-use transfer, found out conversion from barren to crop land being the largest over the region with area approximately 6** × **10^4^ km^2^ to 49** × **10^4^ km^2^ (averaging 36** × **10^4^ km^2^ in all SSPs).
iv. At the end of the century, (2081–2100), the dominant land-use was still barren land, forest land and crop land, the barren land area will occupy an area of approximately 901** × **10^4^ km^2^ to 1509** × **10^4^ km^2^ (averaging 1243** × **10^4^ km^2^) as a reduction of 16% relative to the historical period (1995–2014). The forest land occupies an area of approximately 92** × **10^4^ km^2^ to 332** × **10^4^ km^2^ (averaging 212** × **10^4^ km^2^) denoting a reduction of 38% relative to the historical period (1995–2014). Likewise, the crop land occupies an area of approximately 377** × **10^4^ km^2^ to 674** × **10^4^ km^2^ (averaging 500** × **10^4^ km^2^) denoting an increase of 105% relative to the historical period (1995–2014). In respect to land-use transfer, found out conversion from barren to crop land being the largest over the region with area approximately 2** × **10^4^ km^2^ to 52** × **10^4^ km^2^ (averaging 41** × **10^4^ km^2^ in all SSPs).

However, majority of the human effects on the earth system, including influences and interaction, were responsible for land-use changes^1,2^, and has been altered by humans within the last millennium^9,10^. SSPs are intended to have different environmental implications, while global land-use models differ by design all aim to modelled same global systems capturing same systems dynamics. Our findings are similar with previous studies investigating uncertainties in land use projections. For example^17,50,51^, both founds large differences in land-cover projections between models, with the highest variability occurring in future cropland areas.

Our findings suggests that cropland, grassland, and urban land will increase over time, exceeding barren land, forest land in the region. This will lead to the biggest expansion in agriculture across Africa in future land use. Climate change is a result of the climatic and socioeconomic changes that could affect how agricultural land is used in the future^4^. Our result agrees with^52^, that LULC analysis in Sialkot Pakistan revealed 4.14% increase in the built-up area and 3.43% decrease in vegetation cover of the city during 1989 to 2020. Both land covers are expected to change in the future (year 2030) by + 1.31% (built-up) and − 1.1% (vegetation). The LUH2 datasets are instruments for the future land-use change scenarios^53^, but there are uncertainties associated with these models, especially in relation to the African Monsoon circulation and precipitation^54^, these variations have been attributed primarily to global sea surface temperature (SST)^54^, which coincides with the period of rapid population growth and associated changes in land use^55^. However, African region could be considered as priorities for biodiversity loss, ecosystem disruptions and carbon storage loss, due to projected scenarios show an upwards trend of cropland expansion, all SSPs depict high conversion to cropland except SSP4-3.4 shows a minimal conversion. Furthermore, previous land-use model intercomparison have highlighted uncertainty arising from differences between initial land-use input data, bioenergy production assumptions and yield responses to climate change associated with underlying crop models^14,56,57^. For example^50^, found out that models often allocate land-use change based on land use in adjacent grid cell (e.g. cropland expansion at the edge of existing agricultural area), therefore, can have a large influence on the dynamic of cropland expansion in future time steps^50^.

However, future food demand will likely be met by other means such as crop land expansion or greater reliance on imports which further increase cropland^58^. Our finding was in consistent with the study by^17^ who found out that cropland expansion in Africa is likely to continue in the future, which could have significant impact on biodiversity and carbon storage through loss of biomass and soil carbon^17,59^ in their studies over Africa suggests that cropland is expected to increase by approximately 51% (154 million hectares) from 2020 to 2090 under future land-use climate change scenarios. A future Africa Green Revolution may result in increased agricultural use and CO_2_ emissions; therefore, the expansion of cropland may also be due to growing global market integration^60^. The total food production in Africa will only suffice to feed 1.35 billion people, at a time when the continent's population is expected to reach 3.5 billion, leaving a food deficit for 2.15 billion people^58^. Africa holds just 9% of the world's surface water, while accounting for over 17% of the world's total population^61,62^.

Additionally, the primary driver of the largest conversion of barren land to crop land might also related to regional climate change^1,6^. Similarly, from^63,64^ reported a projected increase in precipitation across Africa from CMIP6 projections indicate enhanced precipitation across many regions under various scenarios, this increase in precipitation will significantly favour the conducive environment for agricultural productivity^65^. Climate change is anticipated to make agricultural development more difficult as cropland in the region increases^66^. Overall, these findings highlight the need for in-depth research into additional concerns, particularly those across continents, especially considering the expansion of cropland within Africa and the impact of climate change.

The region is identified as a climate change hotspot and is experiencing rapid population growth^42^, food insecurity accounted for 811 million globally, including 282 million in Africans (accounted for 21%) are faced by climate related shocks, changes in land tenure and agrarian system of production, high-income inequality and economic downturns worsened by the COVID-19 pandemic^67^.

Although cropland is being expanded to increase agricultural output, this threatens biodiversity and carbon storage, which has an impact on how well ecosystem’s function^68–70^. Only about 19% of African GDP is made up of the agricultural sector^58^. Despite this, Africa is a key hotspot for food insecurity and climate change vulnerability since most of its nations are not currently and will not be able to sustain themselves in the face of a changing environment^38^.

Socioeconomic development shift and population growth, urbanization and economic changes serves as a major driver to land use changes in different regions^24,71^, thereby this study uses the latest population, economic and land use simulation data sets available under various SSPs to project future land use changes and their relationships with socioeconomic changes in Africa in different time scales, the study also performed a relationship between land use simulation and observational land use to identify the performance of the SSPs with observation and was found to be strong linear relationships exist among the two datasets. The SSPs under different development pathways might result in the possibility of future changes to storyline for both socioeconomic and land use variables under different IAMs models scenarios to uncertainties of the projections^72^. Despite the high resolution of LUH2 datasets with multiples crops and pasture types and related management practices still shows uncertainties at some points^7^.

However, our studies found a strong relationship between population and crop land and GDP and crop land in Africa, these also agrees with the previous results regarding changes in socioeconomic level (population and GDP)^73–75^.

Our studies depict that averagely under all SSPs Africa population might reaches 2341.89 million accounted for (72%) increase compared with 2020 and the highest was under SSP3 and GDP in our finding grows to 3031.06 billion USD accounted for increase to (30.31%). These findings corroborated with previously result that SSP2 sees an increase of global average income under a future of global progress where developing countries achieve significant economic growth”^25,76,77^. According to^76^, GDP per capita is projected to increase in all African countries. Meanwhile, croplands constitute 10% of the total land area on the African continent^78^. However, over half (58.4%) of African croplands are located on drylands, where crop production is becoming increasingly difficult due to ‘water shortages, land degradation, climate change and persistent poverty’^78,79^. Under climate change, in wet tropical regions drylands are expected to become wetter, while in Northern and Southern Africa, subtropical drylands will expand, and semi-arid zones may shift to arid or hyper-arid zones^79,80^.

The possible mechanism behind land-use change over Africa could perhaps reflect history linked with economic development, population growth, technology, and environmental changes^81^. The transition from constant to rising rates of land use change in Africa has been discussed in the context of shifting global food regimes and coincides with a period when global food production changed from agro technological intensification (driven by the Green Revolution in the 1960s) to the production for globalized markets and increasing trade, especially during the 1990s^82,83^. For example, higher rates for the changes in land-use in developing countries might be because of demand from developed countries in terms of global economy and international trade being an important agent (driver) in land use change, as affirmed by growing global market integration, changing in the opportunity created by market and outside policy intervention^5,60^. The changes might be in the area or the intensity of use^81^, as affirmed by^71^ future changes in the area will have significant influences on the global economic growth, industrialization, and allocation of resources.

The result of this study has an important implication for policy interventions under climate change as the region is faced with serious climate change issues and extreme events (see AR4 and 5^36,37^). The shift in socioeconomic development indices might result to climate change^24,71^, thereby to develop specific measures to mitigate such will help a long way in the regions. It is important to note that, under a changing climate, identified by land use change and socio-economic development some countries in Africa may not have the resources to export food products and may experience deficits as well while others will have a surplus^38^. This poses an important question that should be further explored. First, affordability, will the region be able afford to import the additional food needed to meet their population's demand despite their growing GDP under climate change. As the region is faced with population growth such research would be crucial in developing efficient short- and long-term plans to combat and adjust to regional and local land-use changes in relation to changing climate and socioeconomic parameters which is the most critical components in preparing and mitigating their consequences towards sustainable land-use management practices.

Future land use change with LUH2 datasets can be incorporated into land policy in several ways over Africa. Here are some possible approaches:

Informing land-use planning: LUH2 datasets can provide valuable information on how land use is likely to change in the future under different scenarios. This information can be used to inform land-use planning decisions, such as where to locate new developments, how to manage natural resources, and how to protect sensitive ecosystems.

Assessing the impact of land-use policies: Land-use policies can have a significant impact on future land use. LUH2 datasets can be used to assess the impact of different land-use policies on future land use patterns. For example, policymakers can use LUH2 datasets to evaluate the impact of zoning regulations, conservation programs, and other land-use policies on future land use.

Identifying areas of high risk: LUH2 datasets can be used to identify areas that are at high risk of future land-use change. This information can be used to prioritize conservation efforts and to target interventions aimed at reducing the impact of land-use change on sensitive ecosystems.

Developing land-use scenarios: LUH2 datasets can be used to develop land-use scenarios that explore different future land-use patterns under different scenarios. These scenarios can be used to inform land-use planning decisions and to evaluate the impact of different land-use policies on future land use.

Overall, incorporating LUH2 datasets into land policy can help policymakers make more informed decisions about how to manage land use in a way that is sustainable and equitable. Therefore, can be used as policy in Africa by providing information on how land use and land cover will change over time, related to land management, conservation, and development. It is recommended to take extensive further research on the cropland exposure on disaster risk management and prioritizing areas in the future.

## Methodology, study area, data and methods

### Study area

**Figure 8:**
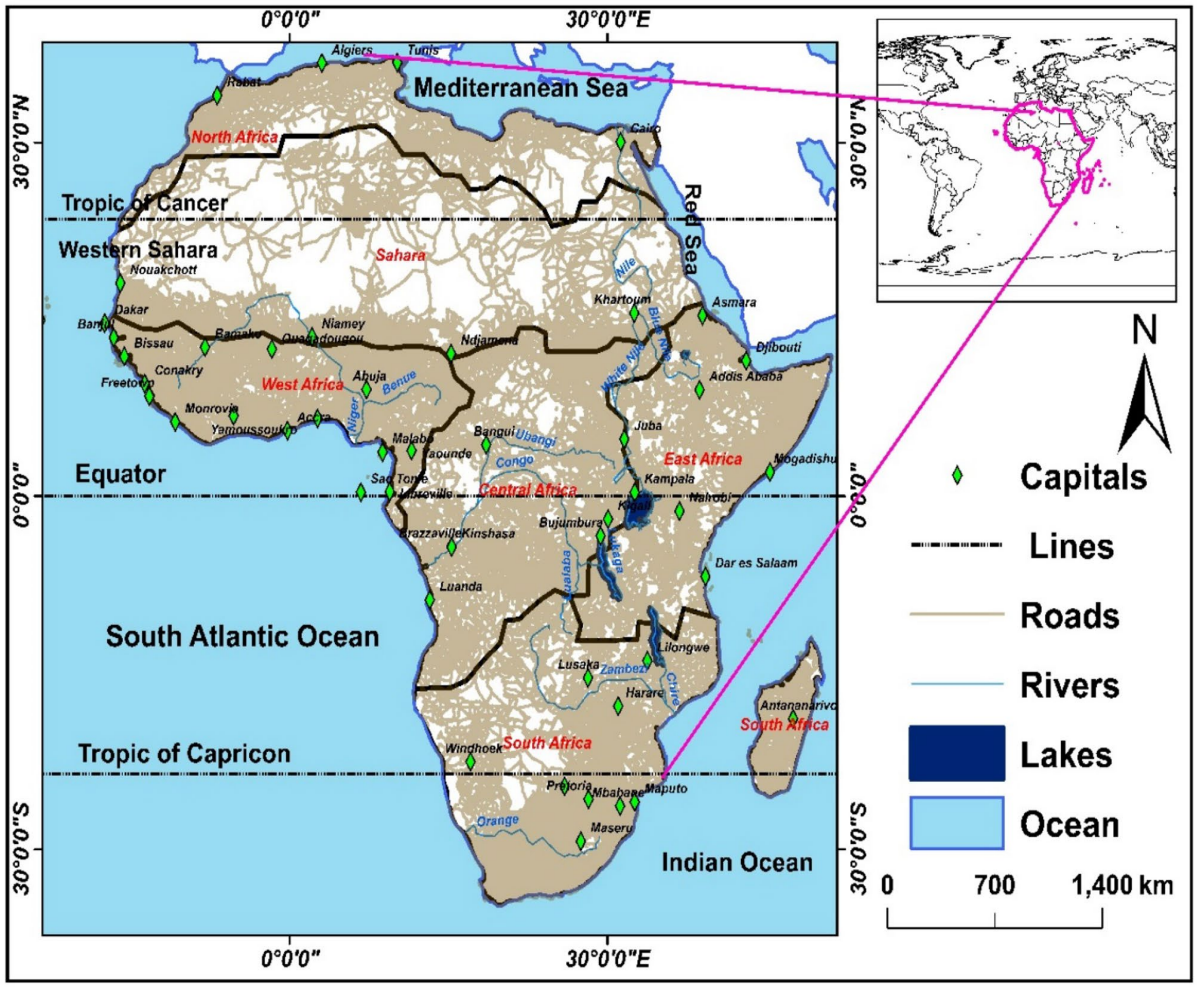
Map of Africa including six selected regions.

We concentrate on Africa and its six sub-regions: Northern Africa (NAF), the Sahara (SAH), Western Africa (WAF), Central Africa (CAF), Eastern Africa (EAF), and Southern Africa (SAF). These regions are selected as recommended by^84^, database https://www.un.org/en, all situated in the tropics^85^, with elevations ranging from 0 to 5895 m, and are geographically located between 32° N and 35° S and 19° W and 52° E. The vast territory of Africa, which covers over 30.37 million km^2^, has a climatologically diverse landscape^86^, Mount Kilimanjaro in Tanzania, which rises 19,340 feet above sea level, is its highest point. At 515 feet below sea level, Lake Assal in Djibouti is where it is at its lowest point (Fig. 8). The second-largest continent in the world, Africa is rich in natural resources including copper, gold, and diamonds. It consists of 53 nations, some of which are landlocked and have no direct access to the sea. Its borders are the Mediterranean Sea to the north, the Atlantic Ocean to the west, the Red Sea to the northeast, and the Indian Ocean to the southeast. The Nile, which travels 4145 km from Burundi to Egypt, is the longest river in Africa (Fig. 8). Lake Victoria, which spans 26,724 square miles and is located between Tanzania, Kenya, and Uganda, is the largest lake. Other notable rivers in Africa include the Senegal River, Niger River, Zambezi River, Orange River, Kasai River, Lualaba River, and Limpopo River. The region is the most tropical of all the continents because it contains both the Tropic of Cancer and the Tropic of Capricorn (Fig. 8).

Africa's average annual precipitation is less than 700 mm year^−1^ and the average temperature is between 15 and 27 °C. According to^87^ the intertropical convergence zone (ITCZ) oscillation modulates the precipitation distribution in space and time, which helps define Africa’s two unique weather patterns. Complex interactions between the vegetation cover and weather and climate are seen^88,89^. Land use and land cover (LULC) changes have been occurring on the continent for many years^90^. According to^89,90^, the period 1980–2005 reflects and captures all forms of anthropogenic activities in the region, particularly in global warming^89,90^.

### Datasets

#### Historical land-use data

Historical land use information was gathered from the ESA World Cover project, gotten from https://worldcover2020.esa.int which uses modified Copernicus Sentinel-1 and -2 data at a resolution of 10 m. This project was built and validated in almost real-time, while also maximising the impact and uptake for the end users. According to^91^, the globe cover product is given in an elliptical WGS 1984 grid with a regular latitude/longitude grid (EPSG:4326). To achieve the research goal, only 2020 datasets were chosen for the study, and regional land use types were reclassified for comparison with future land use data.

#### Future land-use data

The scenarios pathways delineate possible future societal advancements^92^. Notably, the Scenario Model Inter-comparison Project (Scenario-MIP), a significant component of CMIP6, has merged SSPs with RCPs to formulate novel scenarios for simulation and projection experiments^30^. In its initial phase, the Scenario-MIP experiment combined five SSPs (SSP1, SSP2, SSP3, SSP4, and SSP5) with four RCPs (RCP2.6, RCP4.5, RCP6.0, and RCP8.5).

The main objectives of LUH2 project are to produce an integrated set of land use scenarios data from 850 to 2100 linking the historical land use reconstruction to future, projections of land use changes, key agricultural management information and land use shifts at a resolution^7^ of 0.25° × 0.25°. Input from CMIP6 for the period 2015–2100 was based on SSP–RCP scenarios from Integrated Assessment Models (IAMs)^72^. In this study, land-use data from 7 SSPs of 5 Integrated Assessment Models (IAMs) were used to support decision-making by providing insights on global environmental change and sustainable development change that are important to policy. (http://luh.umd.edu/).

In particular, the impacts on the agricultural economy, land use and trade, as well as energy demand and supply, are important to consider when assessing the socio-economic effects of climate change mitigation policies using the Integrated Model to Assess Greenhouse Effect (IMAGE)^93^. Energy, climate, environment, and sustainable development are all examined holistically and cross-cuttingly in the Model for Energy Supply Strategy Alternatives and their General Environment Impact (MESSAGE)^94^. Global climate change and its effects on the context of land and climatic elements are integrated into the Asia Pacific Integrated Model (AIM)^29^. The Global Change Assessment Model (GCAM), another integrated assessment model, integrates the economic, energy, agricultural, and land-use systems with the climate^95,96^. The Regional Model of Investment and Development (REMIND) and Model of Agricultural Production and it Impacts on the Environment (MAgPIE) compose of REMIND-MAgPIE, this integrate assessment model framework, with REMIND integrating a microeconomic growth model and an energy model and MAgPIE being a global multiregional economic land-use optimization model^14,29,97^.

#### Socioeconomic data

This study uses a dataset of population and gross domestic product (GDP) grids under 5 Shared Socioeconomic Pathways (SSP1, SSP2, SSP3, SSP4 and SSP5) (10.57760/sciencedb.01683). The data timescale spans from 2010 to 2100, with the spatial resolution^71,92,98–100^ of 0.5° × 0.5°. The reference population and GDP for 2020 were accessed from https://www.un.org/en, for comparison.

### Methods

#### Reclassification of land-use types

The land use in Africa comprises of six major categories: desert/barren land, built-ups, waterbodies, farmlands/shrubs, grasslands, and forestlands (https://worldcover2020.esa.int). In LUH2, there are 12 types of land use categories, and water is assumed to be constant over time, including the future; therefore, future changes in water are not projected under LUH2^7^. However, Table 3 shows the land use type under LUH2, and the current land use types in Africa and those in LUH2 are unified and reclassified in to five major categories for consistent such as: barren land, cropland, forested land, grassland, and urban land (Table 1)^7^.

**Table 3:** Reclassification of land use types on Africa Region.

Current land use type	Type of land use in the LUH2 Project	Land use type reclassification
Cropland	C3 annual crop	
	C3 perennial crop	
	C4 annual crop	Cropland
	C4 perennial crop	
	C3 nitrogen-fixing crop	
Forest land	Forested primary land	Forest land
	Potentially forested secondary land	
Grassland	Managed pasture	Grassland
	Rangeland	
Construction Land	Urban land	Urban land
	Non-forested primary land	
Unused land	Potentially non-forested secondary land	Barren land
Water	–	–

#### Dynamics land-use transfer process

Dynamic land use transfer involves a process by which allocation of land for various purposes, such as agriculture, industry, and residual usage evolved over time in response to social, economic, and environmental factors^81,101^. These changes are driven by a complex interaction of variables, including population increase, economic development, technological advancement, and changes in land value^71,102^. This process is known as dynamic land use, and it refers to the transfer of land from one designated use to another^103^.

#### Correlation coefficient between observational land use and SSPs scenarios

In this study, we used a Pearson's correlation analysis to show how historical data from the ESA in 2020 and SSPs from the same year performed. These coefficients serve to measure the strength and direction of the association between the two samples (i.e., observation land-use and SSPs land-use). The Pearson correlation coefficient (r) can be calculated using the formula given below:

1 $$r=\frac{n\left(\sum xy\right) - \left(\sum x\right)\left(\sum y\right)}{\sqrt{\left[n\sum {x}^{2} - {\left(\sum x\right)}^{2}\right]\left[n\sum {y}^{2} -{ \left(\sum y\right)}^{2}\right]}}$$

where: r = Pearson Coefficient, n = number of observations for both the land-use historical and SSPs simulations, $\sum xy$ = sum of for both the land-use historical and SSPs simulations, $\sum x$ = sum of x historical land use, $\sum y$ = sum of y SSPs simulation land use, $\sum {x}^{2}$ = sum of historical x, land use, $\sum {y}^{2}$ = sum of SSPs simulation y land use.

**Figure 9:**
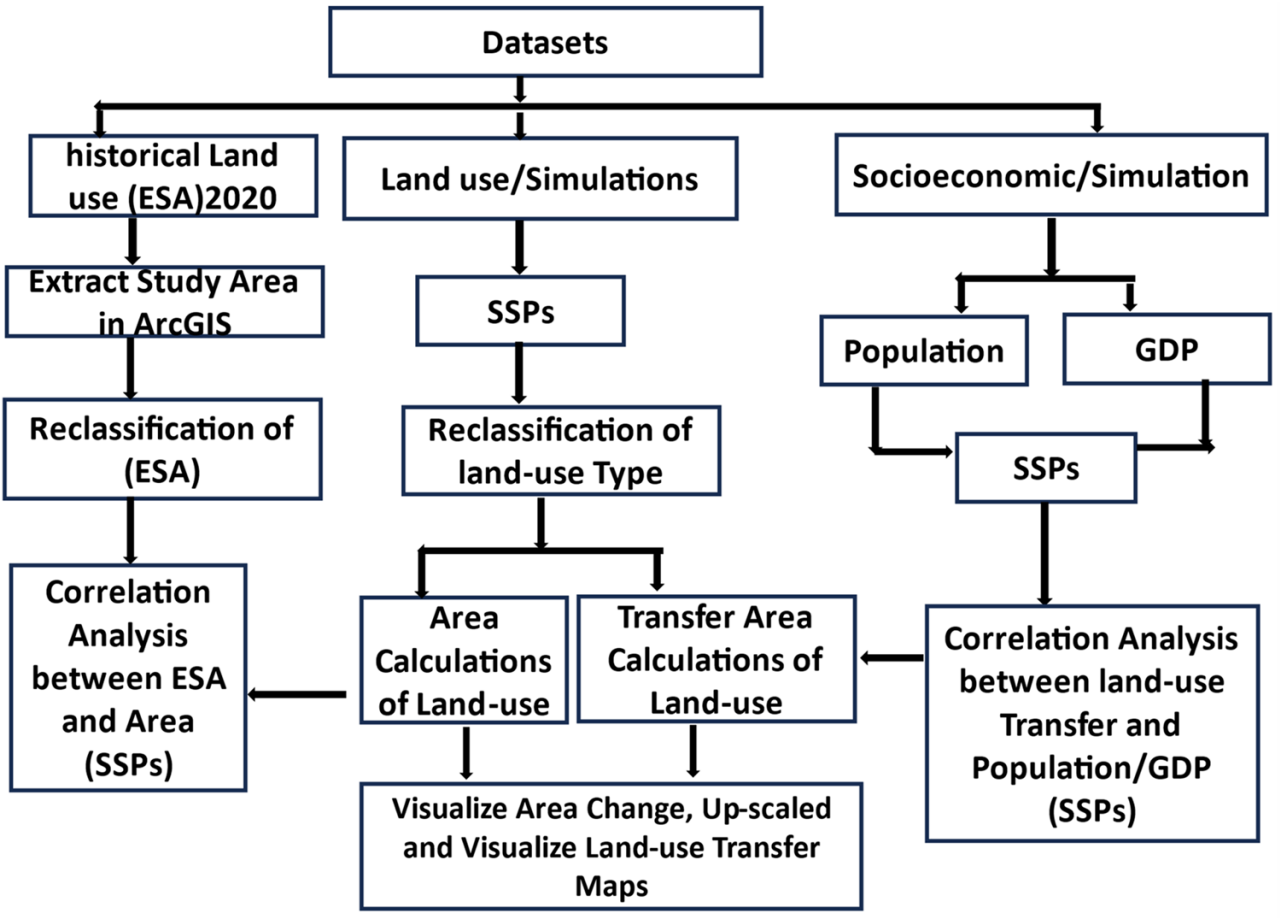
Methodological framework uses in this study.

## Data Availability

We are grateful to ESA World Cover Project for land use output, for making their land use dataset available at https://worldcover2020.esa.int. We are also grateful to Land-use Harmonization 2 (LUH2) website (https://luh.umd.edu/). We are also thankful to Gridded dataset for population and economy under Shared Socioeconomic Pathways [DS/OL]. V1. Science Data Bank, 2022 [2023-10-11] https://cstr.cn/31253.11.sciencedb.01683. We are also grateful to the United Nation population and economy division, New York: World population Prospect at https://www.un.org/en for making their data available.
